# The Role of Artificial Intelligence in the Radiological Diagnosis of Urogynecological and Obstetric Disorders: A Narrative Review

**DOI:** 10.7759/cureus.114707

**Published:** 2026-08-18

**Authors:** Uppalapati Priyanka, Renubala Rout, Neekita Meher, Amar Kumar, Vaibhav Shridharrao Nichat, Ashish Kumar Shukla, Amit Bisht

**Affiliations:** 1 Department of Obstetrics and Gynaecology, All India Institute of Medical Sciences, Mangalagiri, Mangalagiri, IND; 2 Department of Obstetrics and Gynaecology, Krantijyoti Savitribai Phule Brihanmumbai Municipal Corporation Hospital, Brihanmumbai Municipal Corporation, Mumbai, IND; 3 Department of Radiodiagnosis, Institute of Medical Sciences and SUM Hospital, Bhubaneswar, IND; 4 Department of Urology, All India Institute of Medical Sciences, Bibinagar, Bibinagar, IND; 5 Department of Imaging, Super Diagnostic Centre, Amravati, IND; 6 Department of Radiodiagnosis, Santosh Medical College, Santosh Deemed to Be University, Ghaziabad, IND; 7 Department of Radiological Imaging Techniques, College of Paramedical Sciences, Teerthanker Mahaveer University, Moradabad, IND

**Keywords:** artificial intelligence, obstetric imaging, radiology, urogynecology, women’s health

## Abstract

Artificial intelligence (AI) has emerged as a transformative tool in radiological diagnosis, particularly in urogynaecology and obstetric disorders where accurate and timely imaging is essential. This narrative review evaluates current AI applications across key imaging modalities, including ultrasound and magnetic resonance imaging, emphasizing improvements in diagnostic precision, automation, and workflow efficiency. AI-driven approaches such as machine learning, deep learning, and radiomics enable automated segmentation, anomaly detection, and quantitative assessment of conditions, including pelvic floor dysfunction, urinary incontinence, endometriosis, and fetal abnormalities. In obstetric imaging, AI supports automated fetal biometry, anomaly detection, and risk prediction for complications such as preeclampsia and preterm birth. Integration of AI into radiological workflows enhances reporting efficiency and facilitates clinical decision-making through predictive analytics and standardized outputs. Challenges related to data quality, model generalizability, interpretability, and regulatory frameworks continue to limit widespread clinical adoption. Future directions should focus on multicenter datasets, explainable AI, and multimodal integration to improve reliability and clinical translation. Furthermore, strengthening clinician-AI collaboration and incorporating real-world validation studies will be critical for safe implementation. Overall, AI has significant potential to enhance diagnostic accuracy, reduce variability, and support personalized care in women’s imaging.

## Introduction and background

Urogynecological and obstetric disorders are a significant part of the global health problem of women, including pelvic organ prolapse (POP), urinary incontinence, endometriosis, and the entire range of pregnancy-related complications [1]. These conditions have a major impact on the quality of life, reproductive outcomes, and maternal and fetal morbidity and mortality, especially in low- and middle-income contexts in which access to specialized treatment is still low [2]. The growing number of such conditions due to aging, the growing cesarean section rates, and late childbearing have increased the need for quality, timely, and repeatable methods of diagnosis [3]. Radiological imaging is instrumental in the assessment and treatment of urogynecological and obstetric illnesses. Ultrasound is the safest and first-line modality because it is real-time, safe, and widely available, especially in fetal evaluation and pelvic floor evaluation [4]. Magnetic resonance imaging (MRI) offers better soft tissue contrast. It cannot be eliminated in complex cases of deep infiltrating endometriosis, dysfunction of the pelvic floor, and fetal anomalies of the central nervous system [5]. Despite high doses of radiation, which render it an unsuitable technique in obstetrics, computed tomography (CT) may still be useful in certain emergencies like postpartum complications and trauma. Collectively, the imaging modalities are the foundation of the diagnostic processes, and they are used to inform both clinical and treatment planning [6].

These developments have been made, and traditional radiology interpretation has several limitations. Interobserver and intraobserver consistency have also been issues, especially in subtle or complicated cases like early POP or mild fetal anomalies [7]. The standards of diagnosis may also rely on the skill of an individual who performs the test, especially ultrasound, whereby the images received and interpreted are highly reliant on the operator [8]. Moreover, radiologist workload and burnout also increase owing to the increasing imaging research, which can influence the work of a radiologist and turnaround times [2]. The restrictions point to the need for the implementation of aiding technologies, which may raise the degree of accuracy, standardization, and effectiveness in practicing radiology. Machine learning (ML) and deep learning (DL), being types of artificial intelligence (AI), have turned out to be groundbreaking technologies in the medical imaging sphere. AI algorithms, such as convolutional neural networks (CNNs), have proven to be exceptionally good at image recognition, image segmentation, and image classification tasks, and in some cases, perform tasks as effectively as human professionals do [9]. AI systems can be used by radiologists to analyze vast amounts of imaging data, detect intricate patterns, and generate a tangible result that is hard to discern using traditional analysis, all in the interest of deriving benefit [10]. These capabilities make AI a potential instrument in enhancing the workflow of diagnostics and decreasing variability.

AI has been found to have potential applications in automated segmentation of pelvic floor structures, organ descent, and structural analysis for the identification of abnormalities that lead to urinary incontinence and prolapse in the context of urogynecological imaging [11]. Similarly, in obstetric imaging, AI models have been developed for automatic fetal biometry performance, standard planes, and early diagnosis of fetal anomalies, leading to increased consistency and reduced operator dependency [12]. Predictive modeling of the adverse outcomes of pregnancy, which can be preeclampsia and preterm birth, is also being used with advanced applications that enable the integration of imaging data with clinical parameters [13]. Some of the benefits of AI implementation into radiological practice are the increase in diagnostic accuracy, the workflow efficiency, and a decrease in the variability of observers [4]. Moreover, with the help of AI-based radiomics, it is possible to extract high-dimensional quantitative imaging data features that help to characterize a disease and preventive measures against risks that cannot be assessed using traditional visual analysis [14]. Nevertheless, concerns like the heterogeneity of data, the applicability of algorithms, their interpretability, and regulatory issues are to be overcome to provide safe and effective clinical implementation.

Considering the extremely fast development of AI technologies and their growing uses in the imaging of women, an in-depth synthesis of existing data is justified. This narrative review will critically discuss the use of AI in diagnosing radiology of urogynecological and obstetric diseases, its applications today, clinical implications, limitations, and future projections [8]. Combining already available information, this review is aimed at presenting a systematic body of knowledge regarding the implementation of AI in diagnostic imaging and its possible impact on changes in the clinical practice of this particular field [15]. The primary data relevant to AI in both the field of urogynecological ultrasound and obstetric ultrasound are still scattered, and there is no multi-center and extensive validation study that could help to demonstrate the relevance of this technology to different patient populations and image environments. A large number of the models that are available in the market also rely on stand-alone diagnostic exercises and are not linked to real cases and decision-making systems. In addition to these limitations, AI may introduce potential disadvantages into clinical practice. Algorithmic bias resulting from non-representative training datasets may reduce diagnostic reliability across diverse patient populations, while false-positive or false-negative outputs may adversely affect clinical decision-making. Limited interpretability of complex AI models, concerns regarding patient-data privacy and security, uncertainty regarding accountability for AI-assisted diagnostic errors, and excessive dependence on automated outputs may also restrict clinician confidence and safe adoption. The other inadequacies include a lack of standardization of datasets, reporting metrics, and regulatory procedures, which limit more extensive clinical translation.

Objective of the review

This narrative review intends to critically evaluate the role of AI in the radiological diagnosis of urogynecological and obstetric disorders as it is. It will be based on integrating knowledge from the field of existing literature on applications of AI in various imaging modalities and clinical environments. Assessment of the diagnostic performance of these technologies, clinical utility, and limitations will be another objective of the review. Additionally, it will seek to highlight the existing challenges and future directions for AI in clinical practice.

## Review

Fundamentals of AI in radiology

AI in radiology is a broad term encompassing a variety of computational techniques that emulate human cognition, particularly in pattern recognition and decision-making [16]. ML is a subset of AI that is used for systems that learn without direct programming, and DL is a subset of ML that uses a multi-layered neural network that can model complex non-linear relationships in images [10]. These methods can be classified as supervised learning, in which models are learned from labeled data, and unsupervised learning, in which patterns in unlabeled data are uncovered. Supervised learning methods are more frequently used in practice in clinical imaging tasks [17]. Among the different algorithms, CNNs are used with great success in medical imaging because they can automatically create spatial hierarchies of learned features on image data. The primary applications of CNNs in the radiological field are image classification, image detection, and image segmentation [18]. In the biomedical image delineation problem, a U-Net-type network is more suitable for an attempt to define anatomical structures and pathological regions with high accuracy, where fetal and pelvic images are concerned [19]. Besides, radiomics, as another complementary methodology, is also described in detail, which is characterized by the high-dimensional quantitative nature of medical imaging data extracted to provide disease characterization, prognosis, and prediction models [20].

The application of AI in radiology works in a systematic pipeline, but with many steps, such as data acquisition, data pre-processing, model building, model validation, and clinical deployment. To create powerful models, annotated datasets should be high quality, and models can be improved and generalized by applying preprocessing tasks such as normalization and augmentation [2]. Models are then tested on independent datasets after training to find out their diagnostic accuracy and reliability, and are then applied to clinical systems. It needs to integrate smoothly with the current radiology system, including the Picture Archiving and Communication System (PACS), to allow for timely decision-making and optimisation of workflow [20]. The key points and processes of AI in the radiological field have been outlined systematically in Table 1.

**Table 1:** Key Components and Workflow of AI in Radiology PACS: Picture Archiving and Communication System This table was created by the authors based on information synthesized from previously published studies [2,10,16-20].

Component	Description	Clinical Relevance in Radiology	References
Artificial Intelligence (AI)	Broad computational systems simulating human cognition and decision-making	Enhances diagnostic support and pattern recognition	[16]
Machine Learning (ML)	A subset of AI enabling systems to learn from data without explicit programming	Used in predictive modeling and classification tasks	[10]
Deep Learning (DL)	Advanced ML using multilayer neural networks for complex data analysis	Improves image interpretation and automated diagnostics	[10]
Learning Types	Supervised (labeled data) and unsupervised (pattern detection in unlabeled data)	Supervised learning is widely applied in clinical imaging	[17]
Convolutional Neural Networks (CNNs)	Algorithms that learn spatial features from images	Applied in classification, detection, and segmentation tasks	[18]
U-Net Architecture	Specialized DL model for biomedical image segmentation	Enables precise delineation of anatomical structures	[19]
Radiomics	Extraction of quantitative imaging features	Supports disease characterization and prognostic modeling	[20]
AI Workflow Pipeline	Includes acquisition, preprocessing, training, validation, and deployment	Ensures structured and reproducible AI integration	[2]
Data Preprocessing	Techniques such as normalization and augmentation	Improves model performance and generalizability	[2]
Clinical Integration	Integration with PACS and radiology systems	Facilitates real-time decision-making and workflow efficiency	[20]

Imaging modalities in urogynecology and obstetrics

Radiological imaging is an essential part of the diagnostic assessment of urogynecological and obstetric disorders, as it allows assessing both structures and functions of a variety of clinical conditions [21]. Clinical indication, safety, and outcome of the diagnostic selection are used as the basis for selecting the imaging modality, and ultrasound and MRI are the most common tools in everyday use [3]. Ultrasound (2D, 3D, Doppler) has been the primary imaging modality in obstetrics and urogynecology because it is real-time, non-ionizing, and is available universally. In natal ultrasound, two-dimensional (2D) ultrasound is used regularly to measure the biometry of the fetus, its anatomy, and to measure pelvic floor muscles and muscles of the pelvic organs. Three-dimensional (3D) ultrasound offers more accurate spatial visualization and is used in cases of POP and defects in the levator ani muscle [22]. The Doppler ultrasound also helps in the determination of vascular flow, which plays a significant role in the measurement of placental perfusion, fetal status, and gynaecological conditions, including endometriosis and adnexal masses [23]. Ultrasound has several advantages; however, it is still operator-dependent, and the variability in image acquisition and interpretation is the source of inconsistency in diagnosis [6]. MRI has excellent soft tissue contrast and multiplanar imaging, and thus has become a critical necessity in complex urogynecological and obstetric cases [9]. MRI has special applications in the measurement of deep-penetrating endometriosis, deep-lying pelvic floor, and congenital malformation of the uterus, and fetal imaging, where the central nervous system and the thoracoabdominal structure must be thoroughly studied [24]. Diffusion-weighted imaging (DWI) and functional MRI are other advanced MRI techniques that help in better characterization of tissues and diagnostics. In obstetrics, MRI is used as a follow-up to ultrasound when the sonographic results are inconclusive or are limited by factors in the maternal or fetal environment [4].

The application of CT in obstetric imaging is limited because of the exposure of the baby to ionizing radiation. Yet, it is still applicable in certain clinical contexts, especially in the emergency department, including postpartum hemorrhage, trauma, and possible pulmonary embolism, when the need to provide timely and correct diagnosis is crucial [25]. CT can also be applied in urogynecology to evaluate complex pelvic masses or complications that cannot be well characterized by ultrasound or MRI, but it is usually used secondarily to other techniques [26]. New advances in imaging, such as elastography and fusion imaging, are beginning to attract attention due to the possibility of improving the accuracy of the diagnosis. Elastography offers a quantitative evaluation of tissue hardness, which can be effective in describing the disorders of the pelvic floor, uterine abnormalities, and placental pathology [27]. Fusion imaging methods (a combination of real-time ultrasound with already obtained MRI or CT images) make it possible to localize the anatomy of the lesion and its targeted evaluation. When combined with AI frameworks, these advancements can also enhance the overall development of a diagnostic workflow and patient clinical outcomes in the area of women's imaging [28]. All in all, both imaging modalities have their own strengths and weaknesses, and their combination makes it possible to have a complete assessment of urogynecological and obstetric conditions. Combining new imaging technologies with new computing devices is likely to improve diagnostic accuracy and standardization even more in this area [9]. Table 2 shows a summary of imaging modalities and their clinical use in urogynecology and obstetrics.

**Table 2:** Imaging Modalities in Urogynecology and Obstetrics MRI: magnetic resonance imaging; CT: computed tomography; CNS: central nervous system This table was created by the authors based on information synthesized from previously published studies [6,24,25,27,28].

Modality	Key Features	Clinical Applications	Limitations	References
Ultrasound (2D, 3D, Doppler)	Real-time, non-ionizing, widely available	Fetal biometry, anatomical survey, pelvic floor assessment, vascular evaluation	Operator-dependent variability in interpretation	[6]
MRI	High soft tissue contrast, multiplanar imaging	Endometriosis, pelvic floor dysfunction, fetal CNS, and structural anomalies	Cost, limited availability, longer acquisition time	[24]
CT	Rapid imaging, high spatial resolution	Emergency conditions (trauma, hemorrhage, embolism), complex pelvic pathology	Ionizing radiation, limited obstetric use	[25]
Elastography	Measures tissue stiffness	Assessment of pelvic floor disorders, uterine and placental pathology	Limited availability, evolving evidence	[27]
Fusion Imaging	Combines ultrasound with MRI/CT data	Improved lesion localization and targeted assessment	Technical complexity, limited clinical adoption	[28]

AI applications in urogynecological disorders

AI has been used more intensively in urogynecological imaging to improve the accuracy of diagnostic images, automated image inspection, and the interrater reliability of diagnostic images. The applications are especially applicable in scenarios in which minor anatomical and functional pathologies are hard to evaluate through the use of traditional radiology interpretation [9]. Pelvic floor dysfunction is another significant field of AI usage, as automated segmentation methods allow the proper delineation of the pelvic organs, including bladder, uterus, and levator ani muscles [29]. CNNs, as well as other DL models, have been shown to identify and characterize structural defects, such as levator ani avulsion and the descent of pelvic organs, and enhance the consistency of diagnosis [30]. These devices also help in the objective measurement of pelvic floor biomechanics, which is usually variable in the interpretation of the manual [6].

Functional abnormalities that are not easily seen on static images have been demonstrated to be potentially detected by AI-aided analysis of dynamic pelvic imaging in urinary incontinence. ML models could be used to analyze data obtained through temporal imaging to evaluate the mobility of the bladder neck and urethral support, resulting in a greater level of classification and treatment of the incontinence subtypes [31]. The quantification methods based on AI have also been handy in the assessment of POP. Traditional grading techniques are founded on clinical and radiographic impressions, which are subjective, yet ML algorithms may provide objective outcomes of organ descent and compartment involvement [7]. These approaches enable automated staging and increase reproducibility to assist in the process of clinical decision-making and surgical planning [30].

**Figure 1:**
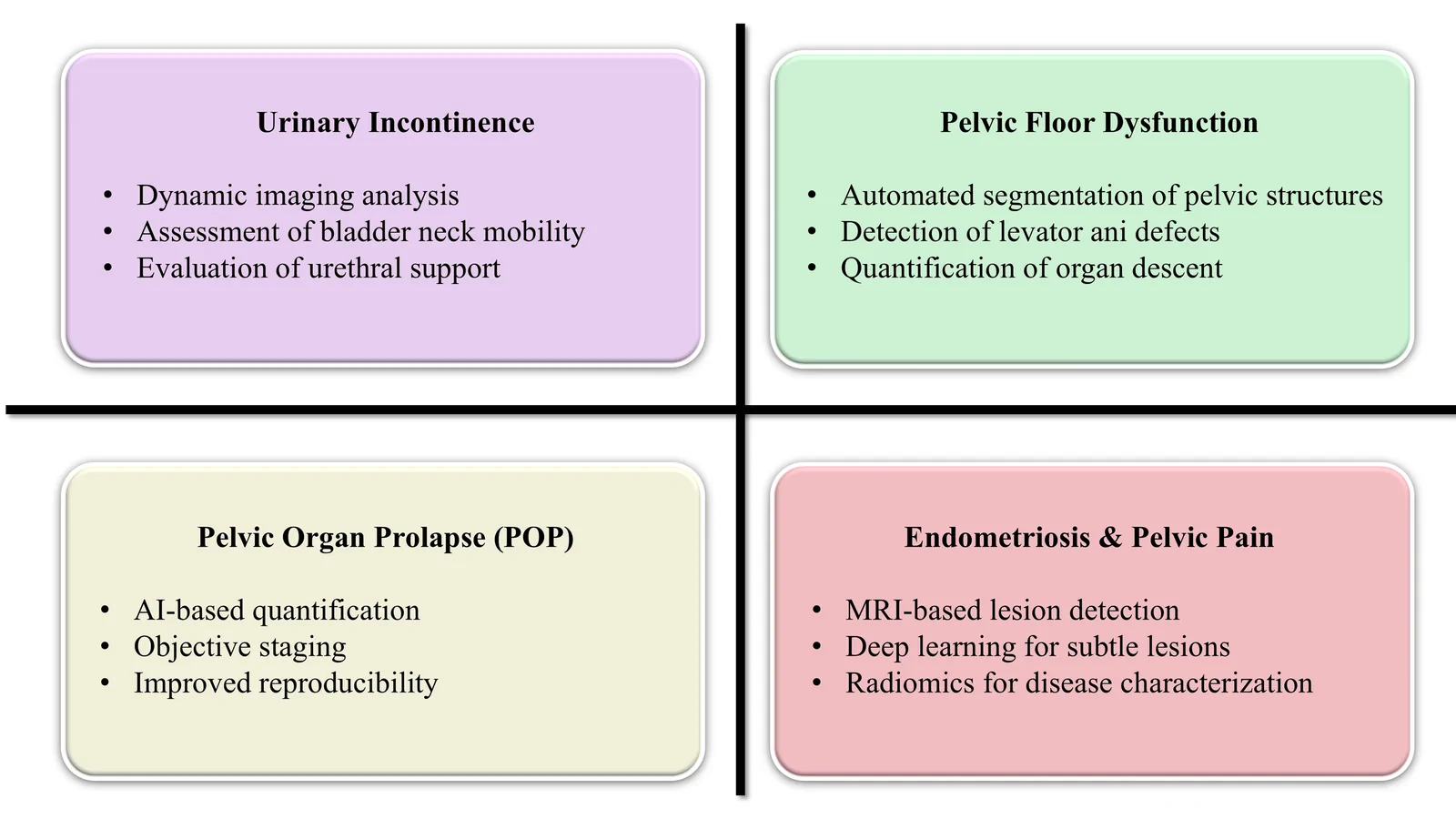
AI Applications in Urogynecological Disorders The figure was created by the authors using Microsoft PowerPoint (Microsoft Corp., Redmond, WA, USA).

In the context of endometriosis or the chronic syndromes of pelvic pain, the use of AI has been directed towards improving the detection and characterisation of lesions, in particular on MRI. The DL algorithms have been more sensitive in identifying deep infiltrating endometriotic lesions, which tend to be challenging to identify due to their varying appearances [32]. Additionally, quantitative imaging features can be extracted using some approaches of radiomics and may be applied to enhance disease phenotyping of diseases and could be employed to predict treatment response [13]. The application of AI to urogynecological imaging is a change that will bring more objective, quantitative, and reproducible diagnostics. This area can tremendously improve the quality of diagnosis and patient outcomes as long as these technologies are being developed and their clinical application is used. Figure 1 shows that AI has the most important applications in urogynecological disorders.

Clinical workflow integration of AI in radiology

The integration of AI in radiological workflow is also changing clinical practice by making the process more efficient and standardized at various stages of the imaging process [33]. AI can also play a role in image acquisition and image pre-processing, whereby automated quality control can optimize image acquisition, reduce artifacts, and standardize imaging protocols in particular cases, like ultrasound, which is operator-dependent [8]. The preprocessing methods (like normalization) and the removal of noise further increase the data consistency and performance of the model [34].

The active introduction of AI in reporting systems is being accomplished through the seamless integration of AI into PACS and Radiology Information System (RIS). These integrations can be used to automatically measure, identify lesions, and create the initial reports, thereby saving on reporting time and increasing efficiency in the workflow [35]. These systems can be beneficial when trying to analyze data in real time and enable radiologists to concentrate on complicated interpretative problems. The AI-based decision-support systems can be used in obstetrics and urogynecological practice to offer diagnostic tips, risk prioritization, and management guidance based on the outcomes of imaging and clinical data [13]. The devices help improve the process of decision-making in the clinical arena, especially in high-risk pregnancies and complex pelvic floor problems.

**Figure 2:**
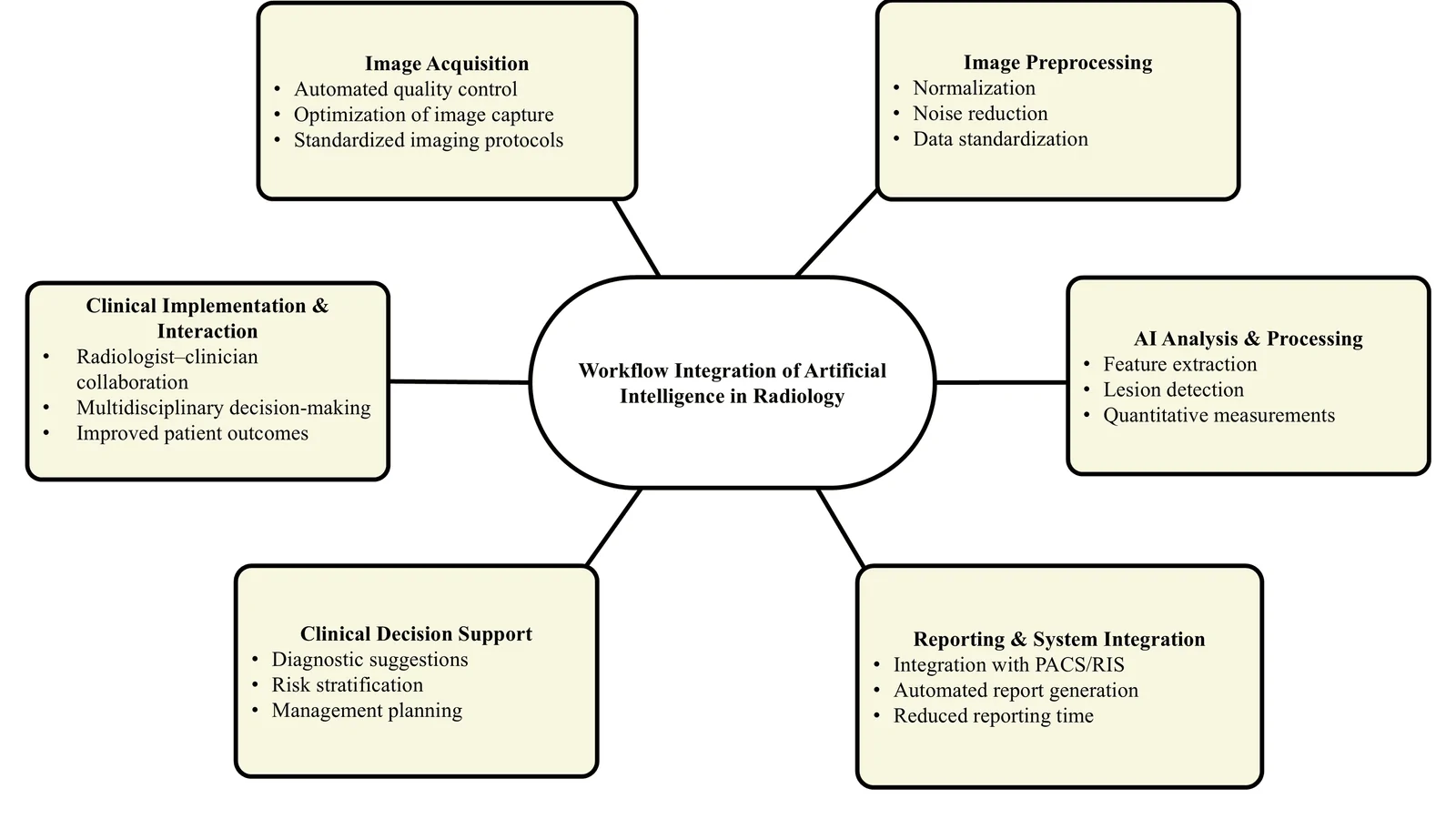
Workflow Integration of AI in Radiology PACS: Picture Archiving and Communication System; RIS: Radiology Information System The figure was created by the authors using Microsoft PowerPoint (Microsoft Corp., Redmond, WA, USA).

Another effect of AI use on radiologist-clinician interaction is the change toward a data-driven and more collaborative practice of patient care [36]. The reason why AI can provide standardized and quantifiable results makes it more efficient in enhancing clarity of communication and making decisions through a multidisciplinary approach, which in the end can produce better patient outcomes [3]. Figure 2 demonstrates that AI may be implemented in the workflow across the different phases of radiological practice.

Data quality, annotation, and model training challenges

The quality and variety of training data are extremely important determinants of the performance and reliability of AI models in radiology. Well-labeled and high-quality datasets are crucial to creating strong algorithms because errors or inconsistencies in labeling might produce a significant impact on the model performance and generalization [37]. With urogynecological and obstetric imaging, such datasets are especially difficult to obtain because of anatomical variation, imaging methods, and manifestation of disease. Pelvic and fetal imaging annotation is further complicated because it involves professional radiological work and is usually time-consuming [32]. Minor anatomic features, fetal movement, and differences in image quality also make accurate labeling challenging and possible interobserver variance even at the annotation phase [6]. These issues will add noise to training data, thus affecting the accuracy of the model.

Bias in datasets and imbalanced classes are also paramount issues to be considered because most AI models train on datasets that might not be sufficient to reflect the diverse population or rare conditions. This may lead to poor performance in its application to other demographic groups or clinical settings, which restricts clinical applicability [38]. To achieve good training and validation of models, there must be standardized procedures, one of which is the use of independent validation as well as an external testing dataset to enhance robustness and reproducibility. The absence of such validation is one of the main drawbacks of a lot of modern research, and stricter methodological frameworks need to be presented in a new study [39]. The main issues of data quality and model training are presented in Table 3.

**Table 3:** Data and Model Training Challenges in AI Radiology AI: artificial intelligence This table was created by the authors based on information synthesized from previously published studies [6,32,37-39].

Challenge	Description	Impact on AI Models	References
Data Quality	Dependence on high-quality, well-annotated datasets	Poor data quality reduces model accuracy and generalizability	[37]
Dataset Variability	Differences in anatomy, imaging techniques, and disease presentation	Limits the consistency and robustness of trained models	[37]
Annotation Complexity	Requires expert input and is time-intensive	Increases cost and time for dataset preparation	[32]
Interobserver Variability	Differences in labeling due to subjective interpretation	Introduces noise and reduces model reliability	[6]
Imaging Challenges	Fetal motion, subtle anatomy, and image quality variations	Affects the precision of annotations and model training	[32]
Dataset Bias	Underrepresentation of diverse populations or rare conditions	Reduces applicability across populations	[38]
Class Imbalance	Unequal distribution of disease vs normal cases	Leads to biased predictions and poor sensitivity	[38]
Lack of External Validation	Limited use of independent datasets for testing	Reduces reproducibility and clinical trust	[39]
Standardization Issues	Absence of uniform training and validation protocols	Hampers the comparison and scalability of models	[39]

Radiomics and predictive analytics

Radiomics has become one of the major developments in the field of medical imaging, allowing the mining of large amounts of quantitative data out of regular radiological images [40]. These characteristics, which comprise texture, shape, intensity, and spatial relationships, provide details that are not provided by visual analysis and enable a detailed description of tissue characteristics and pathological variations [41]. Radiomics enables the objective and reproducible analysis of high-dimensional data by transforming images into high-dimensional data; hence, the analysis is no longer subjective [42].

Radiomics has a role in urogynecological and obstetric imaging in disease phenotyping and risk stratification, where imaging biomarkers related to the severity and progression of diseases are identified. These features can be used in ML models to distinguish benign and pathological states, determine the severity of a disease, including endometriosis or placental malformations, and predict a patient's clinical outcome [43]. This method facilitates personalized medicine as it allows individual management approaches depending on the individual's imaging profile [15]. Also, radiomics, combined with genomic and clinical data, has resulted in advanced predictive models, sometimes called radiogenomics. The models are based on the integration of imaging-obtained characteristics and molecular and clinical data to enhance diagnostic and prognostic evaluation [44]. Such integrative frameworks have the potential to predict complications in early stages in obstetrics, such as preeclampsia and fetal growth restriction, and strengthen preventive and curative interventions [45].

Limitations and future directions

There are limitations to this review. Most of the existing AI evidence in the fields of urogynecology and obstetrics comes from varied studies conducted with different methodologies, small sample sizes, and sparse external validation, and this limited generalizability of the results. The studies also exhibit some variability due to differences in imaging protocols/sets and measures of evaluation. Moreover, most AI systems are black-box systems, which restrict interpretability and clinical trust. Regulatory impediments, privacy of data, and the absence of standardized reporting structures are other factors that hinder the translation into regular practice.

The next directions should involve more focus on the creation of large, multi-centered datasets and unified protocols of imaging and reporting to increase the strength of the models. Explainable AI is required to make progress to enhance transparency and acceptance by clinicians. More accurate predictive analytics might be facilitated with the integration of multimodal information (such as radiological, clinical, and genomic) to improve predictive analytics. Moreover, AI applications in clinical processes and continuous performance assessment should be implemented in real-time and deployed safely, scalably, and effectively. Given the narrative design and heterogeneity of the available studies, no meta-analysis or pooled statistical synthesis was performed, and the evidence was summarized descriptively.

## Conclusions

This review concludes that AI is quickly changing the nature of radiological diagnosis of urogynecological and obstetric disorders and improving accuracy, efficacy, and reproducibility. Implementation of ML and DL algorithms into imaging modalities (ultrasound and MRI) has allowed automatic analysis, better detection of small abnormalities, and normalization of quantitative evaluation. These developments come in handy to minimize operator dependency and interobserver variability, which have been unsolved problems in women's imaging. The application of AI in imaging is being further expanded in radiomics and predictive modeling, to the extent that imaging is being used not only in diagnosis but also in risk stratification and tailored clinical decision-making. Nevertheless, these advantages are not without their challenges, which are associated with heterogeneity of the data, limited validation, interpretability, and regulatory issues that make it not yet universally used by clinicians. These limitations should be overcome by further data standardization, explainable AI, and multimodal integration development. When properly justified and used ethically, AI may become an inseparable component of the usual radiological approach that would possibly improve the results of maternal and gynecological healthcare activities.
